# Effect of Laser Irradiation at Different Wavelengths (940, 808, and 658 nm) on Pressure Ulcer Healing: Results from a Clinical Study

**DOI:** 10.1155/2013/960240

**Published:** 2013-09-12

**Authors:** J. Taradaj, T. Halski, M. Kucharzewski, T. Urbanek, U. Halska, C. Kucio

**Affiliations:** ^1^Department of Physiotherapy Basics, Academy of Physical Education in Katowice, Mikolowska 72 Street, 40-065 Katowice, Poland; ^2^Department of Medical Biophysics, Medical University of Silesia in Katowice, Medykow 18 Street, 40-752 Katowice, Poland; ^3^Department of Physiotherapy, Public Higher Professional Medical School in Opole, Katowicka 68 Street, 40-060 Opole, Poland; ^4^Department of Descriptive and Topographic Anatomy, Medical University of Silesia in Katowice, Jordana 19 Street, 41-808 Zabrze, Poland; ^5^Department of General and Vascular Surgery, Medical University of Silesia in Katowice, Ziolowa 45 Street, 40-635 Katowice, Poland; ^6^Department of Physiotherapy in Internal Medicine, Academy of Physical Education in Katowice, Mikolowska 72 Street, 40-065 Katowice, Poland

## Abstract

The aim of the study was to assess the efficacy of laser therapy (at different wavelengths: 940, 808, and 658 nm) for treating pressure ulcers. The primary endpoint in this trial included both the percentage reduction of the ulcer surface area and the percentage of completely healed wounds after one month of therapy (ulcer healing rate). The secondary endpoint was the ulcer healing rate at the follow-up evaluation (3 months after the end of the study). In total, 72 patients with stage II and III pressure ulcers received laser therapy once daily, 5 times per week for 1 month using a (GaAlAs) diode laser with a maximum output power of 50 mW and continuous radiation emission. Three separate wavelengths were used for the laser treatment: 940 nm (group I), 808 nm (group II), and 658 nm (group III). An average dose of 4 J/cm^2^ was applied. In group IV, a placebo was applied (laser device was turned off). The laser therapy at a wavelength of 658 nm appeared to be effective at healing pressure ulcers. The wavelengths of 808 and 940 nm did not have any effect in our study.

## 1. Introduction

Of the health problems specific to frail older people, pressure ulcers are a major health disorder, and the establishment and spread of an effective treatment method for pressure ulcers are a pressing issue. Pressure ulcers are a common and costly problem in nursing home settings, with prevalence estimates varying widely from 7 to 23% [1]. 

Care and management can have significant economic consequences. Staff time for ongoing assessment, documentation, and dressing changes and expensive pharmaceuticals drain the available resources. Well-documented, promising, and inexpensive methods from alternative medicine are necessary. 

Laser therapy has been used to accelerate wound healing since the late 1960s, but its results are still controversial. The European Pressure Ulcer Advisory Panel (EPUAP) and the National Pressure Ulcer Advisory Panel (NPUAP) published an international guideline, “Treatment of pressure ulcers: quick reference guide,” in 2009 [2]. 

According to the guideline (content titled “Biophysical agents in pressure ulcer management”), several forms of energy have been studied for healing pressure ulcers. These include acoustic, mechanical, and kinetic energy, as well as energy from the electromagnetic spectrum (EMS). Infrared (thermal) radiation and ultraviolet light (invisible light) are all part of the EMS as is electrical/electromagnetic stimulation. The recommendation for biophysical agents is supported by direct scientific evidence from properly designed and implemented clinical studies on pressure ulcers in humans (or on humans at risk for pressure ulcers), providing statistical results that consistently support the recommendation.

One of the proposed physical methods (alternative medicine) in this document is laser irradiation, but the guideline stated that there is insufficient evidence from research on pressure ulcers and other chronic types of wound to recommend the use of laser therapy for treating pressure ulcers. An international collaboration has recently been formed between the National Pressure Ulcer Advisory Panel (NPUAP), the European Pressure Ulcer Advisory Panel (EPUAP), and the Pan Pacific Pressure Injury Alliance (PPPIA), an alliance formed between the Australian Wound Management Association, New Zealand Wound Care Association, Hong Kong Enterostomal Therapy Association, and Wound Healing Society of Singapore. The intent of the collaboration is to develop a 2014 update of the international guidelines for the prevention and treatment of pressure ulcers, which was initially developed by the NPUAP and EPUAP in 2009. Complete clinical information must be received before creating an updated guideline and to discuss the effectiveness of controversial alternative therapies in wound healing.

The aim of this clinical study was to assess the efficacy of laser therapy (different wavelengths—940, 808, and 658 nm) for the treatment of pressure ulcers. The primary study endpoints were the percentage change in the ulcer surface area and in the number of completely healed wounds after one month of therapy (ulcer healing rate). The secondary endpoint was the follow-up ulcer healing rates (3 months after the end of the study).

## 2. Materials and Methods

### 2.1. Settings and Participants

**Figure 1:**
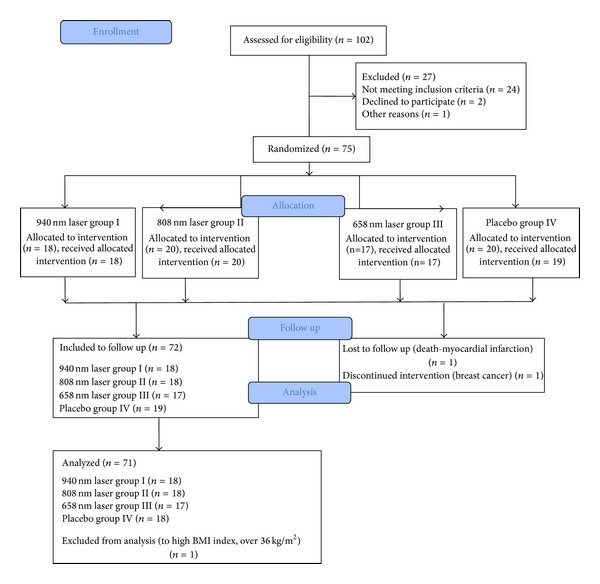
Flow diagram of the study.

The study was performed at the Limf-Med Clinic in Chorzow, Poland, from January 2012 to February 2013. Participating subjects met the following inclusion criteria: (1) presented with a lower extremity pressure ulcer and (2) provided written informed consent to participate in the study. There were no restrictions on gender, race, age, or ulcer duration. Subjects with the following conditions were not allowed to participate or were excluded from the study: (1) clinically detectable infection in the ulcer (critical colonization of bacteria, no signs of healing for two weeks, friable granulation tissue, foul odor, increased pain in the ulcer, increased heat in the tissue around the ulcer, an ominous change in the nature of the wound drainage, e.g., new onset of bloody drainage or purulent drainage, or necrotic tissue in the ulcer); (2) use of drugs, such as corticosteroids, that could interfere with the wound-healing process; (3) use of special dressings, such as hydrocolloids, calcium alginate, activated carbon, or any type of therapeutic procedures different from that used routinely by all groups in the study; (4) nonattendance to the therapeutic program; (5) pregnancy; (6) ankle-brachial pressure index (ABPI) <0.8; (7) diabetes mellitus; (8) systemic sclerosis; (9) cancer diagnosis; and (10) pareses and paralysis caused by injuries to the central or peripheral nervous system. Patients whose pressure ulcers required surgical intervention were also excluded from the study (Figure 1). 

The body mass index (BMI) was calculated for all patients. According to international norms, a BMI higher than 30 kg/m^2^ indicates adipositas. The number of smokers was recorded as well. To determine whether they met the inclusion/exclusion criteria described in the protocol, the recruited participants underwent complex tests (standard blood morphology, immunological studies, HbA_1c_, cholesterol panel, liver enzymes, serum creatinine/glomerular filtration rate, urine testing, and ECG) twice within the three months prior to the experiment. 

### 2.2. Randomization and Intervention

 Participants were randomly allocated to the groups. Computer-generated random numbers were sealed in sequentially numbered envelopes, and the group allocation was independent of the time and person delivering the treatment. The physician (main coordinator) who allocated the patients to groups had 75 envelopes, each containing a piece of paper marked with either group I, II, III, or IV. The physician would select and open an envelope in the presence of a physiotherapist to see the symbol and would then direct the patient to the corresponding group. A clinical nurse collected the data and coded them into an Excel database. The “blinded” results were transferred to a Statistica version 10.0 (StatSoft Inc., Poland) database by a technician. The research coordinators had no contact with and could not identify the patients. 

Subjects from all groups received a routine treatment, including daily simple dressings with sterile gauze after wound cleaning with a 0.9% physiologic solution, use of 1% hydrophilic silver sulfadiazine cream, and an orientation about the use of adapted footwear, self-care, and the prevention of disabilities (the drug therapy was in accordance with the TIME strategy for chronic wound treatment and the EPUAP/NPUAP recommendation from the 2009 reference guide).

Subjects from groups I, II and III received laser therapy once daily, 5 times per week for 1 month. The equipment was a Rainbow Drops (Technomex Group, Poland) gallium-aluminum-arsenide (GaAlAs) diode laser with an output power of 50 mW and continuous radiation emission at separate wavelengths of 940 nm (group I), 808 nm (group II), and 658 nm (group III). The spot size was 0.1 cm^2^. The laser was wired to a cone-shaped applicator (scanner). The laser beam was scanned over the wound surface at a distance of 50 cm from the ulcer surface using a compound movement; the movement frequency was 20 Hz along the ordinate axis and 0.5 Hz along the abscissa axis. The duration of a single procedure was relative to the wound size; the therapy was adjusted to obtain an average dose of 4 J/cm^2^ cm^2^ (direct dose measured on a surface of the wound by Mentor MA10 device, ITAM Inc., Poland). In group IV, the placebo group, a laser was applied in the same manner, but the device was turned off during the treatment sessions (only the applicator was turned on to scan ulcers using noncoherent red visible light). 

The following research was a single-blind, controlled, and randomized clinical trial. The study design, methodology, and treatment doses were programmed by coordinators (physiotherapist, general and vascular surgeons, and an internist). Standard care, infrared camera measurements, and data collection were provided by a nurse. The laser/quasilaser therapy was performed by a physiotherapist. The final statistical analysis was performed by a technician. 

### 2.3. Outcomes Assessment and Follow Up

The ulcer area reduction and the healing rate (number percentage of completely healed ulcers) were evaluated in this study. The healing rate was calculated as the proportion of the number of healed wounds (number of patients) to the total number of patients in that group. The criterion for complete healing was final epithelization of the skin. The nurse and physiotherapist were jointly responsible for deciding whether ulcers were healed. 

**Figure 2:**
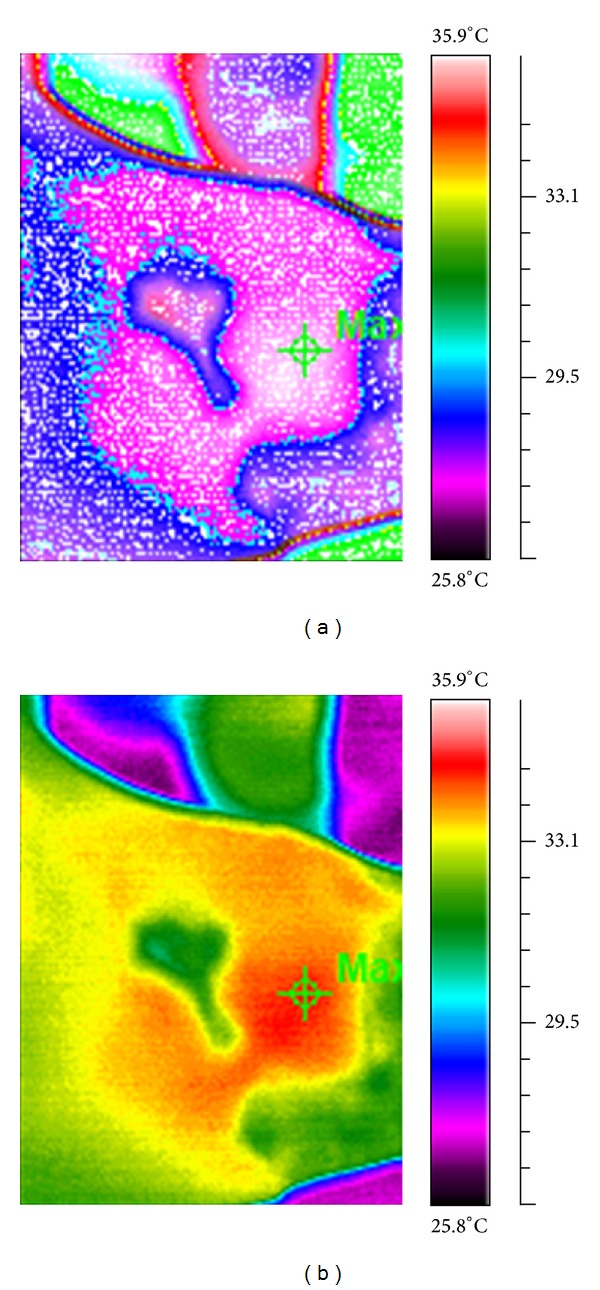
Ulcer area measured using an infrared camera. Example of the healing process of a chronic pressure ulcer under treatment with laser therapy (thermographic image and temperature profile across the ulcer; the difference between warmer/cooler pixels in the wound area was used to calculate the ulcer area).

 A MobIR 3 (Wuhan Guide Infrared Technology, China) infrared camera was used in this study; this uncooled long-wave detector UFPA (third-generation uncooled microbolometer 8 *μ*m–14 *μ*m sensor) has a thermal sensitivity of 80 mK. The infrared camera was connected to a portable computer through a special interface. All images were stored on the computer for further analysis. Images were analyzed using Guide IrAnalyser V1.4 researcher software (Test-Therm Inc., Poland). Infrared thermography was used to monitor the arterial hemodynamic effects on each ulcer (Figure 2). The infrared thermography was based on an analysis of the wound surface temperatures. The reaction is a result of normal or abnormal arterial circulation in capillaries. The measurements were conducted at 24°C. The distance from the camera to the ulcer remained constant (120 cm). The computed images were based on the thermal pixels, which were used to determine the ulcer area (human skin has a thermal emissivity of 0.98 and receives a different color of pixels than open wound). The percentage change of the ulcer area was calculated by a technician as follows.

Δ*S*% is the percentage change of the total surface area (%):

(1) $$\begin{array}{c} \Delta S\%=\frac{\left(S_{I}-S_{F}\right)*100\%}{S_{I}}, \end{array}$$

where *S*_*I*_ is the initial total area (cm^2^) and *S*_*F*_ is the final wound total area after one month (cm^2^). 

The laser therapy (also placebo) in all comparative groups lasted one month. Three months after the therapy, all patients were reviewed (to compare the final healing rates in the groups). During this time, the participants only received routine treatment, including daily simple dressings with sterile gauze after wound cleaning with a 0.9% physiologic solution and use of 1% hydrophilic silver sulfadiazine cream.

### 2.4. Statistical Analysis

To compare the individual parameters that characterized the study groups, the nonparametric Kruskal-Wallis test for countable variables and the chi-squared test (*χ* ^2^) for categorical variables were used. The nonparametric matched pair Wilcoxon test was used to compare the within-group results before and after therapy. The Kruskal-Wallis analysis of variance (*post hoc* Tukey's test) was used to evaluate differences in the percentage changes between the groups (except the healing rates, number of completely healed ulcers, which were compared using the Fisher test). Two-sided results (*P* < 0.05) were considered to be statistically significant.

## 3. Results

In total, 75 individuals were qualified to participate in the treatment. One patient dropped out from the study during therapy in the placebo group (patient chose to discontinue treatment and withdrew from the study for personal reasons, care in the home of her daughter suffering from scarlet fever). Two patients from group II (808 nm laser therapy) had complications unrelated to the treatment and were directed to other hospitals (one patient died) before the follow-up observation. One patient in the control group was excluded from the analysis (BMI over 36 kg/m^2^, which was too high and significantly increased the SD; this increase could have seriously affected the reliability of both the nonparametric Kruskal-Wallis analysis of variance and the final conclusions). 

Of the 71 patients who completed the study protocol, 32 were men and 39 were women, with ages ranging from 24 to 88 years. Twenty participants were obese (fourteen in the laser groups and six in the placebo). The body mass of the other participants was within the normal range. The patients were not addicted to either alcohol or drugs; 31 smoked cigarettes (22 in the laser groups and 9 in the placebo). 

All participants had stage II and stage III pressure ulcers on the EPUAP scale [2] on their lower extremities. The pressure ulcers were located on the legs (33.3% in group I, 27.7% in group II, 35.4% in group III, and 33.3% in group IV), feet (16.6% in group I, 22.3% in group II, 23.5% in group III, and 16.6% in group IV), lateral and medial ankles (22.3% in group I, 22.3% in group II, 17.6% in group III, and 22.3% in group IV), and greater femoral trochanter (27.8% in group I, 27.7% in group II, 23.5% in group III and 27.7% in group IV). 

In the first group (940 nm), four pressure ulcers occurred as a result of poorly fitting orthopedic aids (one case of wrong orthopedic footwear and three prostheses). In five patients, the ulcers developed after mechanical injuries (pressure, abrasion, and scratches), and in another five, temporary immobilization and forced positioning of the body due to surgical intervention (unconsciousness) or multiorgan injuries were considered the causative factors. In two patients, pressure ulcers formed beneath a cast, immobilizing splint, or traction device. Two persons had pressure ulcers because of prolonged inward pressure exerted by plates and screws used for internal bone stabilization. 

In the second group (808 nm), causative factors included immobilization due to postoperative positioning, multiorgan injuries, or being unconscious; poorly fitting prosthetic limbs (three patients) or footwear (one patient); mechanical injury (four); and pressure exerted by plates and screws (two patients) or pressure from a plaster cast (two) or traction device (one). In four patients, pressure ulcers appeared under postoperative dressings. 

In the third group (658 nm), four pressure ulcers occurred as a result of poorly fitting orthopedic aids (two cases of wrong orthopedic footwear and two prostheses). In four patients, the ulcers developed after mechanical injuries (pressure, abrasion, and scratches), and in another five, temporary immobilization and forced positioning of the body due to surgical intervention (unconsciousness) or multi-organ injuries were considered the causative factors. In two patients, pressure ulcers formed beneath a cast, immobilizing splint, or traction device. Two persons had pressure ulcers because of prolonged inward pressure exerted by plates and screws used for internal bone stabilization. 

In the fourth group (placebo), causative factors included immobilization due to postoperative positioning, multi-organ injuries or being unconscious; poorly fitting prosthetic limbs (three patients) or footwear (one patient); mechanical injury (four); and pressure exerted by plates and screws (two patients) or pressure from a plaster cast (one) or traction device (one). In five patients, pressure ulcers appeared under postoperative dressings. 

**Figure 3:**
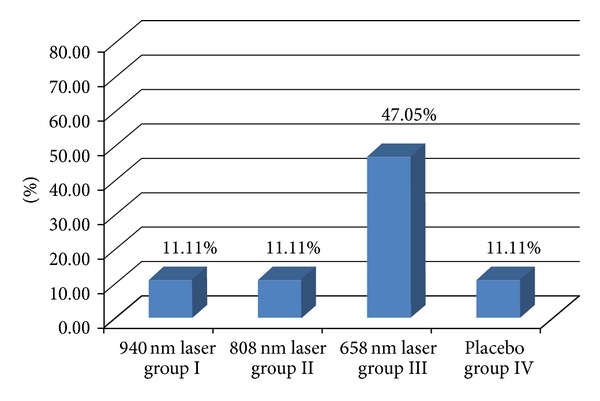
Healing rates (percentage number of completely healed ulcers) after therapy (1-month results). *Fisher test. Groups I and III: 11.11% versus 47.05%, *P* < 0.001, groups II and III: 11.11% versus 47.05%, *P* < 0.001, and groups III and IV: 47.05% versus 11.11%, *P* < 0.001.

**Figure 4:**
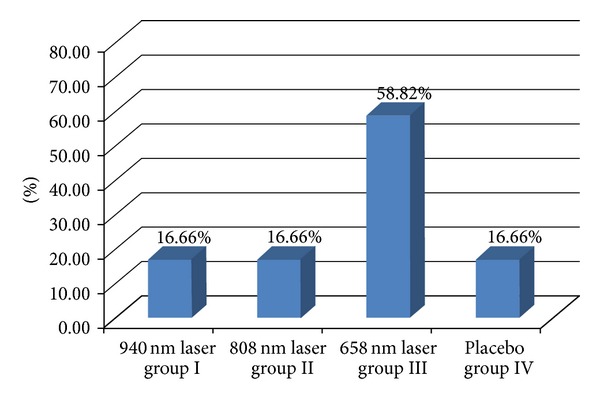
Healing rates (percentage number of completely healed ulcers) at the follow-up analysis (after 3 months). *Fisher test. Groups I and III: 16.66% versus 58.82%, *P* < 0.001, groups II and III: 16.66% versus 58.82%, *P* < 0.001, and groups III and IV: 58.82% versus 16.66%, *P* < 0.001.

The observed groups were homogenous in all participant characteristics (Table 1). After one month of therapy, the healing rate (number percentage of completely healed ulcers) was the highest in group III, 47.05% or 8/17 patients. A significantly worse rate was found in the other groups, only 11.11% or 2/18 patients in each group (Figure 3). Similar results were found in the follow-up analysis (Figure 4).

**Figure 5:**
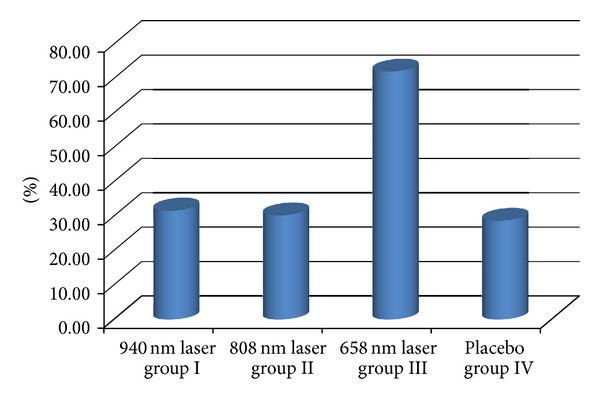
The decrease in the ulcer area after therapy (% of the ulcer area decrease after 1 month). *Kruskal-Wallis test. Groups I and III: 31.23% versus 71.09%, *P* = 0.023, groups II and III: 29.89% versus 71.09%, *P* = 0.018, and groups III and IV: 71.09% versus 28.34%, *P* = 0.011.

 The analysis of the changes of the percentage ulcer area also confirmed that laser irradiation at a wavelength of 658 nm is the most efficient for wound healing. The wavelengths of 940 and 808 nm appeared to be much less effective (Figure 5) and were not better than those in the placebo. 

**Figure 6:**
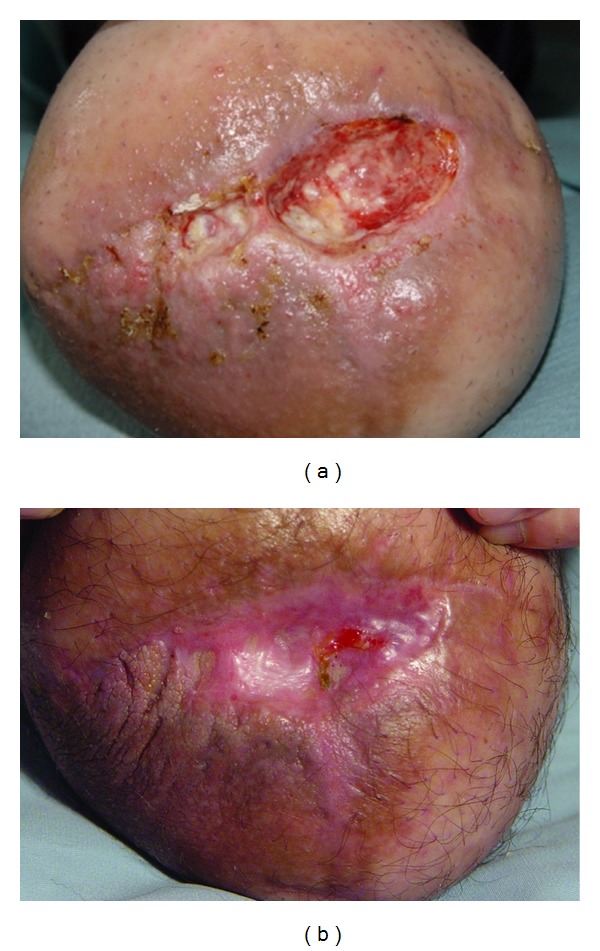
A 49-year-old patient (healing process after 658 nm irradiation) before and after treatment.

**Figure 7:**
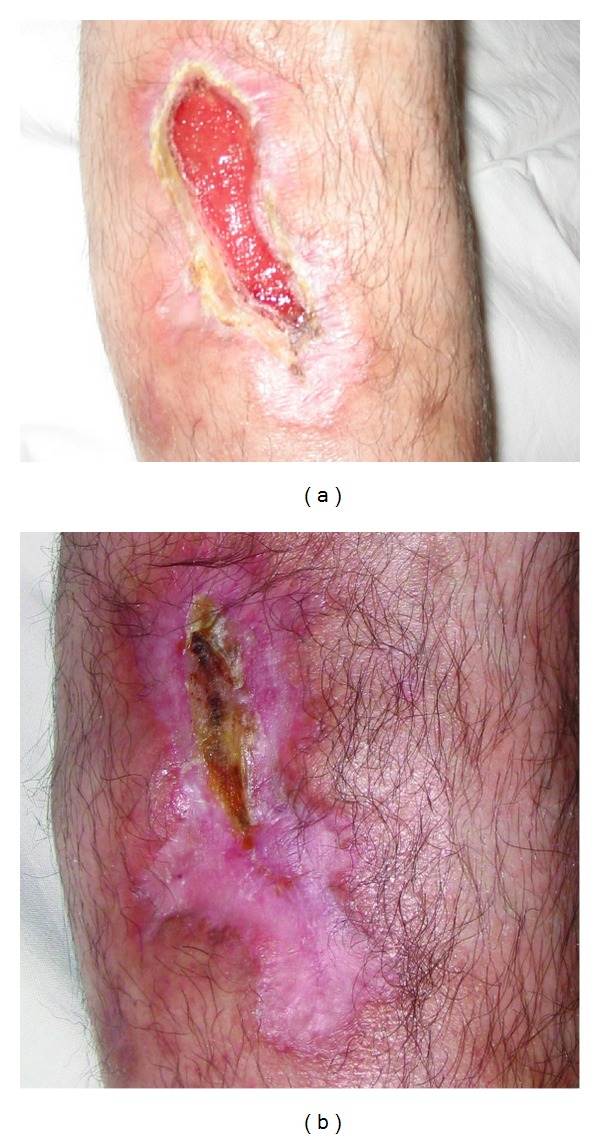
A 53-year-old patient (healing process after 658 nm irradiation) before and after treatment.

Examples of healed ulcers from group III are presented in Figures 6 and 7. The healing process for all groups is shown in Table 2.

## 4. Discussion

Laser therapy has been suggested to be a promising treatment option for sport injuries [3], musculoskeletal disorders [4, 5], neurological problems [6, 7], and open wounds [8]. We only found a few well-documented reports (unfortunately with strongly critical remarks) in the literature about pressure ulcer management. 

For example, Lucas et al. [9] assessed the efficacy of laser therapy (810 nm) in the treatment of stage III pressure ulcers. A total of 86 patients were enrolled in the study. The treatment was the prevailing consensus pressure treatment (*n* = 47); one group (*n* = 39) also received laser therapy, five times a week over a period of 6 weeks. The primary outcome measure was the absolute (mm^2^) and relative (%) wound size reduction at 6 weeks compared with baseline. Secondary outcome measures were the number of patients developing a stage IV ulcer during the study period and the median change in the Norton scores at 6 weeks compared with baseline. The data were analyzed using the intention-to-treat principle and last-observation-carried-forward analyses; the Mann-Whitney *U* tests showed that the differences between the two groups in terms of absolute improvement (*P* = 0.23) and relative improvement (*P* = 0.42) were not significant. During the treatment period, 11% of the patients in the control group and 8% of the patients in the laser therapy group developed a stage IV pressure ulcer (Fisher's exact test: *P* = 0.72). The patients' Norton scores did not change during the treatment period. 

 Taly et al. [10] studied the efficacy of multiwavelength light therapy for treating pressure ulcers. Thirty-five subjects with spinal cord injury, with 64 pressure ulcers (stage 2, *n* = 55; stage 3, *n* = 8; stage 4, *n* = 1), were randomized into treatment and control groups. The treatment group received 14 sessions of multi-wavelength light irradiation, with 46 probes of different wavelengths from a gallium-aluminum-arsenide laser source, 3 times a week. The energy used was 4.5 J/cm^2^. The ulcers in the control group underwent a sham treatment. There was no significant difference in the healing between the treatment and control groups. Eighteen ulcers in the treatment group and 14 in the control group healed completely (*P* = 0.802). The mean time for the ulcers to heal was 2.45 weeks in the treatment group and 1.78 weeks in the control group (*P* = 0.330). 

 Using patients with spinal cord injury, Canadian researchers [11] compared the effect on wound healing of nursing care alone with the effect on wound healing of nursing care combined with either laser treatment or a regimen of ultrasound and ultraviolet C (US/UVC). Twenty patients (22 wounds) were randomly assigned to the treatment groups. All patients received standard wound care consisting of wound cleaning twice daily, application of moist dressings, and continuous relief of pressure until the wounds were healed. The laser protocol consisted of three treatments weekly using a cluster probe with an 820 nm laser diode and 30 superluminous diodes (10 each at 660, 880, and 950 nm) and an energy density of 4 J/cm^2^. The US/UVC regimen consisted of five treatments weekly, alternating the treatment modality daily. The pulsed US was applied at a frequency of 3 MHz and an average spatial-temporal intensity of 0.2 W/cm^2^ (1 : 4 pulse ratio) for 5 minutes per 5 cm^2^ of wound area. The UVC dosage (95% emission at 250 nm) was calculated each session according to the wound appearance. The dosage level was E1 for clean/granulating areas, E3 for purulent/slow-granulating areas, E4 for heavily infected areas, and 2E4 for wound debridement. Wounds were traced every 14 days, and surface areas were calculated using the Sigma-Scan Measurement System. The weekly percentage changes in wound area were compared. The results showed that US/UVC treatment had a greater effect on wound healing than did nursing care, either alone or combined with a laser.

 Some clinical reports are promising. For example, Dehlin et al. [12] included 94 patients (76 patients were evaluated), who were pooled with 87 patients from the earlier study, bringing the total to 163. All patients were treated with monochromatic pulsating light or a placebo over the ulcerated area, according to a specified program for up to 12 weeks. The mean normalized reduction in pressure ulcer size at week 12 was 0.79 for the phototherapy group and 0.50 for the placebo group (95% confidence interval 0.01–0.53; *P* = 0.039). 

In view of the absence of randomized studies with sufficiently large sample sizes, we assessed the efficacy of lasers for treating pressure ulcers. We performed a prospective, single-blinded, and randomized clinical trial to assess the effect of laser therapy as a potential alternative to standard care. We wanted to compare a few common wavelengths in pressure ulcer therapy in a well-prepared and well-planned research program.

The results of our study showed that the wavelength of the laser beam is extremely important during the wound-healing process (and perhaps this is one reason for the many controversies). In this trial, we found no evidence that justifies using laser therapy at wavelengths of 940 and 808 nm as an adjuvant to the future consensus pressure ulcer treatment. However, in our opinion the wavelength of 658 nm is interesting, and its use yielded in promising clinical results. We cannot agree with a general statement that laser therapy does not accelerate the healing process because the correct parameter settings (wavelength, dose, and method of application) must still be demonstrated in the literature. Researchers still do not know all of the physical processes that occur at the cell or tissue level after laser irradiation. We believe that this study will be helpful in preparing the NPUAP/EPUAP 2014 update of the international guidelines for the prevention and treatment of pressure ulcers.

## 5. Weakness of the Study

At this moment, we are able to present the results from a small number of patients and a followup longer than three months (in future, the Kaplan-Meier survival analysis with log rank comparisons is needed). In further studies, we would like to compare the obtained results to those using other colors of lasers, especially blue, which is well known in combination with red light to be highly effective at destroying bacteria.

## 6. Conclusion

Laser therapy at a wavelength of 658 nm appeared to be effective for healing pressure ulcers. The wavelengths of 808 and 940 nm did not have any effect in our study. Future *in vitro* animal and clinical studies are necessary.

## Figures and Tables

**Table 1:** Characteristics of patients.

	Group I	Group II	Group III	Group IV	*P*
Number of patients**	18	18	17	18	>0.05
Age (years)**					
Range	24–88	29–81	32–81	27–70	>0.05
Average	67.39	69.03	68.22	65.34	
Median	69.21	70.11	68.89	66.21	
SD	11.23	12.03	10.09	11.23	
Sex*					
Female	10	10	9	10	>0.05
Male	8	8	8	8	
Adipositas* (BMI > 30 kg/m^2^)	7	7	6	6	>0.05
Smokers*	7	8	7	9	>0.05
EPUAP scale***					
IIA	5	5	4	5	>0.05
IIB	8	8	8	7	
III	5	5	5	6	
Duration of ulcers (months)**					
Range	1.2–3.6	1.3–4.6	1–3.8	2–3.6	>0.05
Average	2.12	2.78	2.03	2.01	
Median	2.02	2.52	2.67	2.39	
SD	2.56	3.01	3.02	2.89	
Initial wound size** (cm^2^)					
Range	0.7–49.6	1.9–49.2	0.9–40.6	0.7–40.1	>0.05
Average	30.23	34.88	32.87	30.89	
Median	34.80	33.99	34.02	29.66	
SD	29.17	36.12	31.33	31.83	

*χ ^2^ test.

**Kruskal-Wallis test.

***Wounds were classified based on the following criteria from the EPUAP scale. Partial-thickness loss of the dermis was considered a stage II ulcer; it was subdivided into stage IIA (shallow lesions involving only the epidermis) and stage IIB (ulcers with damaged dermis). Full-thickness tissue loss was classified as a stage III pressure ulcer and involved subcutaneous tissue and fascia.

**Table 2:** Results in patients with pressure leg ulcers.

	Group	Average ± SD		*P*
		Before therapy	After therapy	
Total ulcer surface area (cm^2^)	I	30.23 ± 29.17	19.23 ± 23.88	0.005
	II	34.88 ± 36.12	21.07 ± 26.02	0.005
	III	32.87 ± 31.33	8.42 ± 14.23	<0.001
	IV	30.89 ± 31.83	20.07 ± 27.23	0.005

*Wilcoxon test.
